# Pancreas Transplant Recipients with and Without Pretransplant Alcohol Use Disorder: A Real-World Cohort Study

**DOI:** 10.3390/medsci14040497

**Published:** 2026-08-19

**Authors:** Arkadeep Dhali, Jyotirmoy Biswas, Sajjad Ahmed Khan, Fayaz Khan, Saikat Mandal, Ashish Sharma, Dushyant Singh Dahiya, Manideepa Maji

**Affiliations:** 1 Academic Unit of Gastroenterology, Sheffield Teaching Hospitals NHS Foundation Trust, Sheffield S10 2JF, UK; 2 School of Medicine, Dentistry and Biomedical Sciences, Queen’s University of Belfast, Belfast BT9 7BL, UK; 3 Department of Medicine, Barasat Government Medical College and Hospital, Barasat 700124, India; 4 Department of Internal Medicine, Birat Medical College Teaching Hospital, Morang 56613, Nepal; 5 Department of Palliative Medicine, Roswell Park Comprehensive Cancer Center, Buffalo, NY 14203, USA; 6 School of Medicine, University of Nottingham, Nottingham NG7 2UH, UK; 7 Department of Hospital Medicine, Yale New Haven Hospital, New Haven, CT 06510, USA; 8 Division of Gastroenterology, Hepatology & Motility, The University of Kansas School of Medicine, Kansas City, KS 66160, USA; 9 Hull York Medical School, University of Hull, Hull HU6 7RX, UK

**Keywords:** pancreas, transplant, alcohol use disorder, real-world study

## Abstract

**Background:** Alcohol use disorder (AUD) is generally regarded as a relative contraindication to pancreas transplantation. Yet the effect of documented AUD before transplantation on long-term results after pancreas transplantation is still poorly understood. Therefore, we used a large real-world electronic health records database to compare the three-year outcomes among pancreas transplant recipients who had and who had not had documented AUD before transplantation. **Methods:** We carried out a retrospective cohort study involving propensity score matching using the TriNetX US Collaborative Network. Each adult patient who had a documented case of alcohol use disorder (ICD-10-CM codes F10.1/F10.2) before transplantation was paired one to one with a patient who did not have such a disorder, matching them on demographic characteristics, comorbidities, transplant-related factors, medications, body mass index, and glycated hemoglobin. The outcomes were evaluated from one day after transplantation up to three years later and comprised all-cause mortality, emergency visits, transplant complications, cachexia, severe protein–calorie malnutrition, pain, vitamin deficiencies, iron deficiency anaemia, diarrhea, and adult failure to thrive. **Results:** Out of 14,367 eligible recipients, propensity score matching resulted in 439 patients in each group. Pretransplant AUD was linked to a significantly higher rate of all-cause mortality (15.1% compared with 8.5%; risk ratio [RR] 1.78, 95% CI 1.22–2.60; hazard ratio [HR] 1.86, 95% CI 1.25–2.78; *p* = 0.002). Cachexia affected 5.2% rather than 2.5% (RR 2.09, 95% CI 1.03–4.24; HR 2.16, 95% CI 1.06–4.44; *p* = 0.036), while diarrhea occurred in 39.6% compared with 28.2% (RR 1.40, 95% CI 1.16–1.69; HR 1.54, 95% CI 1.22–1.94; *p* < 0.001). There were no significant differences in the case of emergency visits (49.0% versus 44.6%; *p* = 0.199), transplant complications (11.1% versus 14.4%; *p* = 0.163), severe protein–calorie malnutrition (12.1% versus 8.9%; *p* = 0.123), pain (59.0% versus 54.7%; *p* = 0.195), vitamin deficiencies (31.4% versus 29.4%; *p* = 0.509), iron deficiency anemia (21.2% versus 21.9%; *p* = 0.805), or adult failure to thrive (7.3% versus 4.3%; *p* = 0.061). **Conclusions:** The presence of a history of alcohol use disorder (AUD) before transplantation was found to be independently linked to a higher three-year mortality rate, as well as the occurrence of cachexia and diarrhea after pancreas transplantation, but it was not associated with an increased number of coded transplant complications or most of the other adverse outcomes following the procedure. These results indicate that pancreas transplant recipients with a history of AUD should have enhanced monitoring in the areas of nutrition, the gastrointestinal system, and addiction.

## 1. Introduction

Pancreas transplantation can restore endogenous insulin secretion and sustained normoglycemia in carefully selected people with insulin-dependent diabetes. Contemporary registry data demonstrate substantial improvements in patient and graft survival, with one-year patient survival exceeding 96% across the principal pancreas transplant categories [1,2]. Nevertheless, transplantation entails major surgery, lifelong immunosuppression, and continued risks of graft dysfunction, infection, cardiovascular disease, and treatment-related morbidity. Careful recipient selection and long-term management of potentially modifiable risks therefore remain central to successful outcomes [2,3].

Alcohol use disorder (AUD) presents a clinically important but insufficiently quantified challenge in this setting. Continued harmful alcohol use is commonly treated as a relative contraindication to pancreas transplantation because of concerns regarding medical comorbidity, psychosocial stability, and adherence to immunosuppressive therapy and follow-up [3,4]. However, a documented history of AUD is not equivalent to active alcohol consumption at transplantation. Determining whether such a diagnosis identifies persistent post-transplant risk is therefore important both for individualized clinical surveillance and for avoiding unsupported exclusion of otherwise suitable candidates.

Evidence from other solid organ transplant populations is limited and inconsistent. In a United States registry study of 60,523 kidney transplant recipients, alcohol dependency recorded at the onset of end-stage kidney disease was associated with higher risks of death-censored graft failure and recipient death [5]. In contrast, a prospective multicenter cohort of 907 kidney transplant recipients found no association between self-reported pretransplant or post-transplant alcohol consumption and graft failure, acute rejection, cardiovascular events, or all-cause mortality [6]. These differing findings highlight the importance of distinguishing a diagnosed disorder from alcohol consumption while accounting for selection effects, coexisting illness, and behavioral risk. Pancreas-specific comparative evidence remains notably sparse.

Several features of pancreas transplant care make this evidence gap clinically relevant. Recipients may already have pancreatitis-related disease, exocrine pancreatic insufficiency, or nutritional vulnerability, while AUD is associated with micronutrient deficiency and altered gastrointestinal motility [7,8]. Gastrointestinal toxicity is also common with transplant immunosuppression; in simultaneous pancreas–kidney recipients, mycophenolate exposure has been associated with acute non-infectious diarrhea [9]. The combined effects of these factors could plausibly influence nutritional morbidity, health care use, and survival after transplantation, but this has not been adequately evaluated in large real-world pancreas transplant cohorts.

Even though poor outcomes linked with alcohol use disorder have been reported in patients who have had kidney or liver transplants, the majority of the existing evidence has been concentrated on these solid organ transplant cases, with relatively little information available concerning those who have had a pancreas transplant, although they have particular metabolic, gastrointestinal, and nutritional weaknesses. We therefore used a large federated United States electronic health record network to compare three-year outcomes among pancreas transplant recipients with and without a documented pretransplant AUD diagnosis. As far as we know, this study is one of the rare large-scale real-world evaluations that specifically looks at whether a documented history of AUD is linked to different post-pancreas-transplant outcomes. Using propensity score matching, we evaluated all-cause mortality, emergency visits, transplant complications, pain, and a range of gastrointestinal and nutritional outcomes. We hypothesized that documented pretransplant AUD would identify recipients at increased risk of mortality and post-transplant gastrointestinal or nutritional morbidity.

## 2. Methods

### 2.1. Study Design and Data Source

We conducted a retrospective, propensity-score-matched cohort analysis using the TriNetX US Collaborative Network, a federated electronic health record network containing diagnoses, procedures, medications, and laboratory data.

### 2.2. Study Population and Exposure Definition

Adults aged at least 18 years were eligible. Pancreas transplantation was identified by any of the following: ICD-10-PCS 0FYG0Z0 (transplantation of pancreas, allogeneic, open approach), ICD-10-PCS 0FYG0Z1 (transplantation of pancreas, syngeneic, open approach), CPT 48554 (transplantation of pancreatic allograft), or ICD-10-CM Z94.83 (pancreas transplant status).

The exposed cohort comprised pancreas transplant recipients with a documented diagnosis of alcohol abuse (ICD-10-CM F10.1) or alcohol dependence (ICD-10-CM F10.2) on or before the pancreas-transplant-defining event. The comparator cohort comprised pancreas transplant recipients without either F10.1 or F10.2 documented on or before the transplant-defining event. Accordingly, the comparator definition excluded documented pretransplant alcohol use disorder but did not explicitly exclude a new alcohol-related diagnosis after the index date.

The index date was the date on which a patient first satisfied the selected cohort criteria. Because the transplant definition included both operative procedure codes and the pancreas transplant status code Z94.83, the index date is the first recorded date satisfying the composite transplant definition rather than necessarily the date of the transplant operation.

### 2.3. Follow-Up and Outcomes

Outcomes were assessed from 1 day to 1095 days after the index date. The platform limited eligible index events to those occurring within the preceding 20 years. The prespecified outcomes were (1) all-cause mortality; (2) emergency visits; (3) transplant complication/rejection, defined by ICD-10-CM T86.89 (complications of other transplanted tissue); (4) cachexia, ICD-10-CM R64; (5) severe protein–calorie malnutrition, ICD-10-CM E43; (6) a pain composite comprising ICD-10-CM R10, G89, or R52; (7) a vitamin deficiency composite comprising ICD-10-CM E55, E56, E51, E52, D51, or D52; (8) iron deficiency anemia, ICD-10-CM D50; (9) diarrhea, ICD-10-CM R19.7; and (10) adult failure to thrive, ICD-10-CM R62.7.

For all-cause mortality and the T86.89-defined transplant complication/rejection outcome, patients with the corresponding outcome before the start of follow-up were excluded from that outcome-specific analysis. For emergency visits, cachexia, severe protein–calorie malnutrition, pain, vitamin deficiencies, iron deficiency anemia, diarrhea, and failure to thrive, patients with a previous record of the outcome were retained. Therefore, these latter analyses estimate post-index occurrence during follow-up and not strictly incident outcomes.

### 2.4. Propensity Score Matching

Propensity score matching was performed using age; sex; race categories; essential hypertension; chronic kidney disease; chronic ischemic heart disease; heart failure; atrial fibrillation/flutter; chronic obstructive pulmonary disease; lipid disorders; tobacco use; type 1, type 2, and other specified diabetes; pancreatic steatorrhea; exocrine pancreatic insufficiency; acute and chronic pancreatitis; acquired partial absence of the pancreas; cachexia; severe protein–calorie malnutrition; vitamin D and other vitamin deficiencies; iron deficiency anemia; long-term insulin use; opioid-, cannabis-, and nicotine-related disorders; insulin, tacrolimus, cyclosporine, mycophenolate mofetil, azathioprine, sirolimus, basiliximab, alemtuzumab, and systemic corticosteroid use; body mass index; and hemoglobin A1c. Covariate balance was assessed using standardized differences. For numeric covariates with missing values (e.g., body mass index, hemoglobin A1c), the TriNetX built-in matching algorithm automatically excluded patients with missing data for any matching variable that was specified in the propensity score model, resulting in a final matched cohort comprising only patients with complete data for the specified matching variables.

Notably, propensity score matching was carried out in order to limit baseline differences between the pretransplantation patients with AUD and without AUD based on a set of predetermined characteristics, comorbidities, and laboratory data, which included the value of HbA1c. Post matching, risk comparisons were performed regarding the predetermined clinical outcomes, and Kaplan–Meier survival analysis was performed to estimate the time-to-event outcomes, reporting HR with 95% CI. Even though HbA1c was included in the propensity score matching, there was still a residual difference between the matched groups. No further multivariable Cox proportional hazards or logistic regression analysis adjusting specifically for HbA1c post-matching was conducted because the database could not support such an adjustment.

### 2.5. Statistical Analysis

Outcome risks were calculated as the number of patients with the outcome divided by the outcome-specific analytic cohort. Between-group estimates included the risk difference, risk ratio, and odds ratio, each with a 95% confidence interval; z statistics and *p* values were reported for risk differences. Time-to-event analyses used Kaplan–Meier estimation. Patients were censored after the last recorded fact in their electronic health record. Between-group curves were compared using the log-rank test, and hazard ratios with 95% confidence intervals were estimated. The platform also reported a proportionality test for each hazard ratio. Median event-free time was reported when the Kaplan–Meier curve fell below 50%; otherwise, it was not estimable within 1095 days. Due to the fact that 10 clinically pre-specified endpoints were assessed, there was a need to take into account the possibility of inflation of the type I error probability of the family-wise error rate due to multiple testing. A significance level of *p* < 0.005 (0.05/10) based on the Bonferroni correction was adopted as an overly conservative sensitivity check, and the false discovery rate was also controlled using the Benjamini–Hochberg method at 5%.

## 3. Results

### 3.1. Cohort Formation and Follow-Up

Our study identified 471 pancreas transplant recipients with documented pretransplant alcohol use disorder and 13,896 recipients without documented pretransplant alcohol use disorder. Propensity score matching included 469 and 13,780 patients, respectively, before matching and yielded 439 patients in each matched cohort (Table 1). Before matching, mean follow-up was 827.050 ± 379.503 days in the alcohol use disorder cohort and 885.859 ± 362.281 days in the comparator cohort; median follow-up was 1095 days in both groups, with interquartile ranges of 602 and 319 days, respectively. After matching, mean follow-up was 834.339 ± 376.915 and 877.442 ± 363.821 days, respectively. Median follow-up remained 1095 days in both cohorts, with interquartile ranges of 575 and 357 days.

### 3.2. Baseline Characteristics

Before matching, the alcohol use disorder cohort was slightly older (49.5 ± 11.9 versus 48.0 ± 12.9 years; standardized difference 0.122), more frequently male (69.5% versus 55.4%; standardized difference 0.293), and more frequently White (77.3% versus 69.5%; standardized difference 0.178). Substantial imbalances were also present across cardiometabolic disease, pancreatitis-related conditions, substance-related diagnoses, immunosuppressive medication use, and laboratory variables. The largest reported standardized differences included nicotine dependence (0.895), systemic corticosteroid use (0.769), insulin medication use (0.700), hypertension (0.653), acute pancreatitis (0.611), chronic pancreatitis (0.592), type 2 diabetes (0.578), long-term insulin use (0.548), tobacco use (0.547), and lipid disorders (0.544).

After matching, the cohorts were similar in age (49.6 ± 12.0 versus 49.2 ± 13.4 years), female sex (30.5% versus 32.1%), and White race (77.2% versus 79.0%). Most listed diagnoses, medication exposures, and body mass index achieved standardized differences below 0.10. Examples included hypertension (74.9% versus 76.3%; standardized difference 0.032), chronic kidney disease (65.1% versus 65.6%; 0.010), chronic ischemic heart disease (32.8% in both groups; <0.001), type 2 diabetes (67.9% versus 71.3%; 0.074), type 1 diabetes (47.4% in both groups; <0.001), acute pancreatitis (24.6% versus 25.5%; 0.021), chronic pancreatitis (23.7% versus 23.2%; 0.011), exocrine pancreatic insufficiency (13.0% versus 13.7%; 0.020), nicotine dependence (37.6% versus 36.7%; 0.019), tacrolimus use (46.7% versus 50.1%; 0.068), mycophenolate mofetil use (35.3% versus 36.7%; 0.028), systemic corticosteroid use (74.3% versus 77.0%; 0.064), and body mass index (25.8 ± 5.7 versus 26.3 ± 5.3 kg/m^2^; 0.082). Hemoglobin A1c remained imbalanced after matching (6.7 ± 1.9% versus 7.2 ± 1.8%; standardized difference 0.264; *p* = 0.001).

### 3.3. Clinical Outcomes

All-cause mortality occurred in 66 out of 438 patients (15.1%) in the alcohol use disorder cohort and 37 out of 437 patients (8.5%) in the comparator cohort (Table 2 and Table 3). The absolute risk difference was 0.066 (95% CI 0.024 to 0.108; *p* = 0.002), the risk ratio was 1.780 (95% CI 1.217 to 2.603), and the odds ratio was 1.918 (95% CI 1.252 to 2.938). At 1095 days, estimated overall survival was 83.05% and 90.32%, respectively. The mortality curves differed by the log-rank test (*p* = 0.002), and the hazard ratio was 1.862 (95% CI 1.245 to 2.784); the reported proportionality-test *p* value was 0.435.

Cachexia was recorded in 23 out of 439 patients (5.2%) with pretransplant alcohol use disorder and 11 out of 439 patients (2.5%) without documented pretransplant alcohol use disorder. The risk difference was 0.027 (95% CI 0.002 to 0.053; *p* = 0.036), the risk ratio was 2.091 (95% CI 1.032 to 4.237), and the odds ratio was 2.151 (95% CI 1.036 to 4.469). The 1095-day cachexia-free probabilities were 93.62% and 97.20%, respectively; the log-rank *p* value was 0.031, and the hazard ratio was 2.164 (95% CI 1.055 to 4.440).

Diarrhea was recorded in 174 out of 439 patients (39.6%) in the alcohol use disorder cohort and 124 of 439 patients (28.2%) in the comparator cohort. The risk difference was 0.114 (95% CI 0.052 to 0.176; *p* < 0.001), the risk ratio was 1.403 (95% CI 1.162 to 1.694), and the odds ratio was 1.668 (95% CI 1.258 to 2.212). The 1095-day diarrhea-free probabilities were 53.29% and 68.04%, respectively. The log-rank *p* value was <0.001, and the hazard ratio was 1.536 (95% CI 1.220 to 1.935); the proportionality-test *p* value was 0.060.

Emergency visits occurred in 49.0% versus 44.6% of patients (risk ratio 1.097, 95% CI 0.952 to 1.263; risk-difference *p* = 0.199). Median emergency-visit-free time was 759 versus 1080 days, and the hazard ratio was 1.146 (95% CI 0.944 to 1.390; log-rank *p* = 0.168). The T86.89-defined transplant complication/rejection outcome occurred in 11.1% versus 14.4% of outcome-eligible patients (risk ratio 0.770, 95% CI 0.532 to 1.114; *p* = 0.163), with a hazard ratio of 0.775 (95% CI 0.523 to 1.150; log-rank *p* = 0.205).

Severe protein–calorie malnutrition occurred in 12.1% versus 8.9% (risk ratio 1.359, 95% CI 0.918 to 2.011; *p* = 0.123; hazard ratio 1.404, 95% CI 0.928 to 2.123; log-rank *p* = 0.106). The pain composite occurred in 59.0% versus 54.7% (risk ratio 1.079, 95% CI 0.961 to 1.211; *p* = 0.195); median pain-free time was 290 versus 459 days, and the hazard ratio was 1.162 (95% CI 0.974 to 1.385; log-rank *p* = 0.093). Vitamin deficiencies occurred in 31.4% versus 29.4% (risk ratio 1.070, 95% CI 0.876 to 1.307; *p* = 0.509; hazard ratio 1.104, 95% CI 0.868 to 1.403; log-rank *p* = 0.421). Iron deficiency anemia occurred in 21.2% versus 21.9% (risk ratio 0.969, 95% CI 0.752 to 1.247; *p* = 0.805; hazard ratio 0.992, 95% CI 0.746 to 1.319; log-rank *p* = 0.954). Adult failure to thrive occurred in 7.3% versus 4.3% (risk ratio 1.684, 95% CI 0.970 to 2.925; *p* = 0.061); the hazard ratio was 1.750 (95% CI 0.992 to 3.088), with a log-rank *p* value of 0.050.

## 4. Discussion

In this multicenter real-world cohort, pancreas transplant recipients with a documented pretransplant alcohol use disorder (AUD) had substantially higher three-year mortality than propensity-score-matched recipients without documented pretransplant AUD. The AUD cohort had a 78% higher risk of death and an 86% higher mortality hazard. Cachexia was approximately twice as frequent, and diarrhea was 40% more frequent. In contrast, there were no clear between-group differences in emergency visits, the T86.89-defined transplant complication/rejection outcome, severe protein–calorie malnutrition, pain, vitamin deficiency, or iron deficiency anemia. Adult failure to thrive showed a borderline signal. Overall, the observed excess was concentrated in survival and gastrointestinal or nutritional morbidity rather than across all measured post-transplant outcomes.

Given that ten prespecified clinical outcomes were analyzed in this study, the problem of multiple comparisons and the risk of committing a type I error is possible. Thus, in order to overcome this concern, we used the conservative criterion of significance based on the Bonferroni adjustment method (*p* < 0.005, 0.05/10) and performed the Benjamini–Hochberg procedure in order to control the false discovery rate at 5%. After adjustment for multiple comparisons, only two outcomes, mortality (*p* = 0.002) and diarrhea (*p* < 0.001), remained statistically significant, and this supports their reliability in the context of multiple comparisons adjustment. At the same time, the association with cachexia (*p* = 0.036) became insignificant after adjustment using the Bonferroni method, and the other outcomes became equally insignificant after adjustment. Thus, only mortality and diarrhea can be seen as more reliable results of the study, while the association with cachexia is a rather tentative result. Importantly, because there were more than one secondary endpoints analyzed, the *p*-values need to be cautiously interpreted. When applying the Bonferroni adjustment to 10 pre-defined clinical endpoints, only all-cause mortality (*p* = 0.002) and diarrhea (*p* < 0.001) proved to be statistically significant, while the relationship with cachexia (*p* = 0.036) was not. Similarly, when applying the Benjamini–Hochberg false discovery rate adjustment, only all-cause mortality and diarrhea were statistically significant, while all other outcomes failed to achieve the statistical significance level. Therefore, the findings most strongly supported by this analysis include the relationships between pretransplant AUD and all-cause mortality as well as diarrhea, and the association with cachexia may be considered a hypothesis-generating result.

The mortality finding is directionally consistent with the United States kidney transplant registry analysis by Gueye and colleagues, in which pretransplant alcohol dependency was associated with higher recipient mortality and death-censored graft failure [5]. It differs from the prospective multicenter study by Jung and colleagues, which found no association between self-reported alcohol consumption and major kidney transplant outcomes [6]. These studies did not measure the same exposure: a coded AUD diagnosis may identify a history of more severe alcohol-related and psychosocial burden, whereas reported consumption may include lower-risk drinking. Differences in recipient selection, exposure timing, and residual confounding may also contribute. The present results extend this concern to pancreas transplantation, but they cannot determine whether active drinking, relapse, or the consequences of previous AUD account for the association. It is important to note that diagnostic coding by itself cannot prove that alcohol exposure was continuing at the time of transplantation or during the follow-up period. Because both a patient who had a past alcohol use disorder and is now in sustained remission and a patient who is currently using alcohol or has recurrent alcohol use can be included in the same diagnostic category, this may weaken or mask the relationship between current alcohol use and the outcomes. Thus, the lack of data regarding the length of remission, the patient’s response to treatment, relapse episodes, and the amount or frequency of alcohol use constitutes a significant limitation when interpreting the association with mortality.

The accompanying gastrointestinal and nutritional findings are clinically coherent but not mechanistically specific. AUD is associated with nutritional deficiency and altered gastrointestinal motility [7,8], while mycophenolate-related diarrhea is well-recognized after simultaneous pancreas–kidney transplantation [9]. In solid organ transplant recipients, diarrhea may also reflect infection or other medication toxicity and can lead to dehydration, altered immunosuppressant exposure, and rejection [10]. Persistent diarrhea has been associated with increased tacrolimus bioavailability [11], while mycophenolate-related gastrointestinal complications may prompt dose alterations with potential implications for transplant outcomes [12]. A large kidney transplant cohort further associated post-transplant diarrhea with graft loss and mortality [13]. In the present analysis, measured immunosuppressive exposures were broadly balanced after matching, and the coded transplant complication outcome was not increased. The diarrhea and cachexia signals may therefore mark a combined burden of gastrointestinal, nutritional, infectious, medication-related, and behavioral vulnerability; the data do not support attribution to alcohol or a specific treatment, nor do they establish that these outcomes mediated mortality.

A number of factors could reasonably account for the increased mortality seen without an equivalent increase in the outcome of transplant-related complications that were coded. The AUD indicator may serve as a marker of more general comorbidity, nutritional vulnerability, infectious episodes, cardiovascular risk, psychosocial instability, or disengagement from ongoing health care management that are not captured through the pancreas-specific complication indicator. In addition, repeated exposure to alcohol, nutritional deficits, or noncompliance with medication can account for complications that result in higher mortality but do not get coded as rejection or transplant failure. It should be noted, however, that such factors are speculative due to the lack of detailed data in the database.

The absence of a difference in the T86.89-defined outcome should also be interpreted with restraint. This code denotes complications of other transplanted tissue and is not an adjudicated measure of pancreas rejection or graft failure. Consequently, the mortality difference cannot be assumed to be independent of graft dysfunction, and it cannot be assigned to rejection, infection, cardiovascular disease, recurrent alcohol use, or any other specific cause. Similarly, the null findings for emergency visits and several nutritional outcomes do not exclude clinically important differences that were uncoded, managed outside of participating organizations, or too infrequent for precise estimation.

These findings should inform risk stratification and follow-up rather than categorical exclusion from transplantation. Although the relapse literature is dominated by liver transplantation, it identifies social support and the stability of pretransplant abstinence as relevant components of risk assessment [14]. A pretransplant AUD diagnosis should therefore prompt a structured assessment of current alcohol use, duration and stability of remission, relapse risk, engagement with addiction treatment, medication adherence, mental health, and social support; standardized tools may improve the consistency of this psychosocial evaluation [15]. Because immunosuppressant nonadherence is associated with rejection and graft loss in transplant populations [16], adherence support should remain a longitudinal clinical priority. After transplantation, closer multidisciplinary follow-up may reasonably include serial weight and dietary assessment, early investigation of diarrhea, review of hydration and electrolytes, nutritional testing when clinically indicated, and monitoring of immunosuppressant exposure during gastrointestinal illness. These actions are clinically plausible responses to the observed risk pattern; their effectiveness requires prospective evaluation.

The study has several strengths. It used a large, multicenter United States electronic health record network, included substantially more pancreas transplant recipients than would usually be available at a single center, and applied one-to-one propensity score matching across a broad set of demographic, clinical, medication, and laboratory characteristics. Agreement between risk-based estimates and time-to-event analyses strengthens the internal consistency of the main mortality, cachexia, and diarrhea signals.

Several important limitations need to be acknowledged. One notable flaw in this research is the possible missing BMI and HbA1c information from the real-life EHR database, which might result in selection bias and residual confounding. Another limitation of the study is that alcohol consumption post-transplantation or relapse cannot be reliably measured using the current database. The presence of pretransplant F10.1/F10.2 does not mean that the patient continues consuming alcohol after the transplantation procedure. On the other hand, a diagnosis of F10.x post-transplantation may indicate repeated recording of the same preexisting condition without a relapse taking place. Also, no information on the quantity of alcohol consumed, whether a person has gone into remission, how many times a relapse has occurred, or involvement in substance abuse treatment programs can be gathered. Therefore, relapse of alcohol post-transplantation and development of a new AUD could not be identified. A further limitation that must be considered is the issue of temporal misclassification of the transplant index date. This can occur because the index date was based on the first date on which either of the two indicators (operative codes or pancreatic transplant status Z94.83) occurred. The index date may not therefore represent the date of transplantation in cases where the transplant status indicator code was assigned weeks or even months after the procedure, and early post-transplant events, such as adverse events or deaths, that occurred prior to assignment of the code were not captured. The issue of temporal misclassification may lead to survival/immortal time bias. Also, adult failure to thrive (R62.7) is a restriction because it is a nonspecific clinical coding concept that is not pathophysiologically diagnosed. In patients who have undergone pancreas transplantation, this can cover several factors, such as malnutrition, frailty or sarcopenia, chronic GI symptoms, depression, deconditioning, social isolation, or problems with self-care. Additionally, the bordering statistical results (log-rank *p* = 0.050; risk difference *p* = 0.061) must be interpreted with caution. Hence, this relationship must be considered only as a possible clinical red flag and not as proof of an adverse event, especially considering that the data base does not differentiate contributing factors to failure to thrive. Similarly, AUD, transplantation, and outcomes were identified from electronic health record codes, making misclassification and incomplete capture likely. The exposure did not distinguish active use from sustained remission or describe severity, quantity, timing, relapse, or treatment. Because the transplant definition included a status code, the index date may not always represent the operation date; the comparator also could develop a new alcohol-related diagnosis after index. Except for mortality and T86.89, previous occurrences of the studied outcomes were not excluded, so post-index events were not necessarily incident. Selection into transplantation may have produced survivor and selection bias. Although matching improved balance, residual confounding remained possible, and glycated hemoglobin remained imbalanced. The data did not reliably specify transplant configuration, donor and operative factors, graft function or loss, immunosuppressant levels and adherence, infection, ongoing alcohol exposure, or cause of death. Finally, some events were uncommon; the cachexia estimate, whose confidence interval was close to the null, is particularly vulnerable to imprecision. These associations are therefore hypothesis-generating and should not be interpreted as causal.

## 5. Conclusions

Among propensity-score-matched pancreas transplant recipients, documented pretransplant AUD was associated with higher three-year all-cause mortality and greater occurrence of cachexia and diarrhea without a clear increase in coded transplant complications or rejection. A history of AUD may identify recipients who benefit from integrated addiction, nutritional, and gastrointestinal surveillance, but it should not be used alone to infer causality or transplant unsuitability. Prospective studies using validated measures of AUD activity and recovery, together with transplant type, graft outcomes, adherence, and cause-specific mortality, are needed to confirm the findings and identify modifiable pathways.

## Figures and Tables

**Table 1:** Baseline characteristics after propensity score matching. *Values are n (%) unless otherwise stated. AUD, alcohol use disorder; SMD, standardized mean difference.*

Characteristic	Pretransplant AUD (*n* = 439)	No Documented Pretransplant AUD (*n* = 439)	SMD
**Demographics**			
**Age at index, years**	49.6 ± 12.0	49.2 ± 13.4	0.031
**White**	339 (77.2%)	347 (79.0%)	0.044
**American Indian or Alaska Native**	10 (2.3%)	10 (2.3%)	<0.001
**Unknown race**	15 (3.4%)	22 (5.0%)	0.079
**Female**	134 (30.5%)	141 (32.1%)	0.034
**Native Hawaiian or Other Pacific Islander**	10 (2.3%)	10 (2.3%)	<0.001
**Black or African American**	61 (13.9%)	50 (11.4%)	0.075
**Male**	305 (69.5%)	298 (67.9%)	0.034
**Other race**	14 (3.2%)	14 (3.2%)	<0.001
**Asian**	10 (2.3%)	10 (2.3%)	<0.001
**Diagnoses and clinical characteristics**			
**Essential hypertension**	329 (74.9%)	335 (76.3%)	0.032
**Chronic kidney disease**	286 (65.1%)	288 (65.6%)	0.010
**Chronic ischemic heart disease**	144 (32.8%)	144 (32.8%)	<0.001
**Heart failure**	80 (18.2%)	78 (17.8%)	0.012
**Atrial fibrillation and flutter**	45 (10.3%)	44 (10.0%)	0.008
**Other chronic obstructive pulmonary disease**	44 (10.0%)	50 (11.4%)	0.044
**Disorders of lipoprotein metabolism and other lipidemias**	256 (58.3%)	274 (62.4%)	0.084
**Tobacco use**	68 (15.5%)	68 (15.5%)	<0.001
**Type 2 diabetes mellitus**	298 (67.9%)	313 (71.3%)	0.074
**Pancreatic steatorrhea**	10 (2.3%)	10 (2.3%)	<0.001
**Exocrine pancreatic insufficiency**	57 (13.0%)	60 (13.7%)	0.020
**Acute pancreatitis**	108 (24.6%)	112 (25.5%)	0.021
**Other chronic pancreatitis**	104 (23.7%)	102 (23.2%)	0.011
**Acquired partial absence of pancreas**	22 (5.0%)	26 (5.9%)	0.040
**Cachexia**	17 (3.9%)	12 (2.7%)	0.064
**Severe protein–calorie malnutrition**	55 (12.5%)	45 (10.3%)	0.072
**Vitamin D deficiency**	130 (29.6%)	118 (26.9%)	0.061
**Other vitamin deficiencies**	11 (2.5%)	14 (3.2%)	0.041
**Iron deficiency anemia**	112 (25.5%)	115 (26.2%)	0.016
**Type 1 diabetes mellitus**	208 (47.4%)	208 (47.4%)	<0.001
**Other specified diabetes mellitus**	128 (29.2%)	134 (30.5%)	0.030
**Long-term current insulin use**	213 (48.5%)	215 (49.0%)	0.009
**Opioid-related disorders**	65 (14.8%)	57 (13.0%)	0.053
**Cannabis-related disorders**	45 (10.3%)	37 (8.4%)	0.063
**Nicotine dependence**	165 (37.6%)	161 (36.7%)	0.019
**Medications**			
**Insulin**	296 (67.4%)	311 (70.8%)	0.074
**Tacrolimus**	205 (46.7%)	220 (50.1%)	0.068
**Cyclosporine**	16 (3.6%)	12 (2.7%)	0.052
**Mycophenolate mofetil**	155 (35.3%)	161 (36.7%)	0.028
**Azathioprine**	21 (4.8%)	17 (3.9%)	0.045
**Sirolimus**	20 (4.6%)	21 (4.8%)	0.011
**Basiliximab**	18 (4.1%)	18 (4.1%)	<0.001
**Alemtuzumab**	11 (2.5%)	12 (2.7%)	0.014
**Systemic corticosteroids**	326 (74.3%)	338 (77.0%)	0.064
**Laboratory/measurement variables**			
**Body mass index, kg/m^2^**	25.8 ± 5.7	26.3 ± 5.3	0.082
**Hemoglobin A1c, %**	6.7 ± 1.9	7.2 ± 1.8	0.264

**Table 2:** Three-year risk analyses in the matched cohorts. *Risk difference, risk ratio, and odds ratio are shown with 95% confidence intervals. The p value is for the risk difference. AUD, alcohol use disorder.*

Outcome	AUD N	AUD Events	Comp. N	Comparator Events	Risk Difference	Risk Ratio	Odds Ratio	*p*
**All-cause mortality**	438	66 (15.1%)	437	37 (8.5%)	0.066 (0.024 to 0.108)	1.780 (1.217 to 2.603)	1.918 (1.252 to 2.938)	0.002
**Emergency visits**	439	215 (49.0%)	439	196 (44.6%)	0.043 (−0.023 to 0.109)	1.097 (0.952 to 1.263)	1.190 (0.913 to 1.552)	0.199
**Transplant complication/rejection**	388	43 (11.1%)	403	58 (14.4%)	−0.033 (−0.079 to 0.013)	0.770 (0.532 to 1.114)	0.741 (0.486 to 1.130)	0.163
**Cachexia**	439	23 (5.2%)	439	11 (2.5%)	0.027 (0.002 to 0.053)	2.091 (1.032 to 4.237)	2.151 (1.036 to 4.469)	0.036
**Severe protein–calorie malnutrition**	439	53 (12.1%)	439	39 (8.9%)	0.032 (−0.009 to 0.072)	1.359 (0.918 to 2.011)	1.408 (0.910 to 2.179)	0.123
**Pain composite**	439	259 (59.0%)	439	240 (54.7%)	0.043 (−0.022 to 0.109)	1.079 (0.961 to 1.211)	1.193 (0.913 to 1.559)	0.195
**Vitamin deficiency composite**	439	138 (31.4%)	439	129 (29.4%)	0.021 (−0.040 to 0.081)	1.070 (0.876 to 1.307)	1.102 (0.826 to 1.469)	0.509
**Iron deficiency anemia**	439	93 (21.2%)	439	96 (21.9%)	−0.007 (−0.061 to 0.048)	0.969 (0.752 to 1.247)	0.960 (0.696 to 1.325)	0.805
**Diarrhea**	439	174 (39.6%)	439	124 (28.2%)	0.114 (0.052 to 0.176)	1.403 (1.162 to 1.694)	1.668 (1.258 to 2.212)	<0.001
**Adult failure to thrive**	439	32 (7.3%)	439	19 (4.3%)	0.030 (−0.001 to 0.060)	1.684 (0.970 to 2.925)	1.738 (0.969 to 3.116)	0.061

**Table 3:** Kaplan–Meier and hazard ratio analyses. *Values separated by a slash are pretransplant AUD cohort/comparator cohort. Event-free probability refers to freedom from the stated outcome, except for all-cause mortality, for which it is overall survival. PH, proportional hazards. AUD= Alcohol use disorders.*

Outcome	AUD Events/N	Comparator Events/N	Median Event-Free Days	1095-Day Event-Free Probability	Hazard Ratio (95% CI)	Log-Rank *p*	PH Test *p*
**All-cause mortality**	66/438	37/437	--/--	83.05%/90.32%	1.862 (1.245 to 2.784)	0.002	0.435
**Emergency visits**	215/439	196/439	759/1080	43.46%/49.84%	1.146 (0.944 to 1.390)	0.168	0.302
**Transplant complication/rejection**	43/388	58/403	--/--	87.09%/83.91%	0.775 (0.523 to 1.150)	0.205	0.592
**Cachexia**	23/439	11/439	--/--	93.62%/97.20%	2.164 (1.055 to 4.440)	0.031	0.195
**Severe protein–calorie malnutrition**	53/439	39/439	--/--	85.88%/90.09%	1.404 (0.928 to 2.123)	0.106	0.211
**Pain composite**	259/439	240/439	290/459	34.57%/40.40%	1.162 (0.974 to 1.385)	0.093	0.704
**Vitamin deficiency composite**	138/439	129/439	--/--	62.53%/66.97%	1.104 (0.868 to 1.403)	0.421	0.068
**Iron deficiency anemia**	93/439	96/439	--/--	75.34%/75.22%	0.992 (0.746 to 1.319)	0.954	0.925
**Diarrhea**	174/439	124/439	--/--	53.29%/68.04%	1.536 (1.220 to 1.935)	<0.001	0.060
**Adult failure to thrive**	32/439	19/439	--/--	91.25%/95.04%	1.750 (0.992 to 3.088)	0.050	0.411

Footnote: --/-- refers to the fact that the median could not be calculated because the Kaplan–Meier survival curve did not drop below 50%.

## Data Availability

The original contributions presented in this study are included in the article. Further inquiries can be directed to the corresponding author.
